# Exploring the spatial covariance of cerebral vascular density and amyloid burden in Alzheimer's disease

**DOI:** 10.1002/dad2.70390

**Published:** 2026-06-18

**Authors:** Lucia Argenti, Alessio Cirone, Federico Massa, Elena Sentieri, Mattia Losa, Luigi Lorenzini, Sara Garbarino, Luca Sofia, Stefano Raffa, Matteo Bauckneht, Gianmario Sambuceti, Wendy Kreshpa, Giulia Bozzo, Giulia Tomassini, Francesca De Cesari, Virginia Pelagotti, Martina Pulze, Lorenzo Gualco, Mehrnaz Hamedani, Chiara Razzetta, Andrea Brugnolo, Nicola Girtler, Stefano Caneva, Pietro Mattioli, Silvia Morbelli, Shahzad Ali, Michele Piana, Carlo Serrati, Antonio Uccelli, Andrea Chincarini, Dario Arnaldi, Luca Roccatagliata, Fabio Bandini, Beatrice Orso, Matteo Pardini

**Affiliations:** ^1^ Department of Neuroscience, Rehabilitation, Ophthalmology, Genetics, Maternal and Child Health (DINOGMI) University of Genoa Genoa Liguria Italy; ^2^ IRCCS Azienda Ospedaliera Metropolitana Genoa Liguria Italy; ^3^ Life Science Computational laboratory (LISCOMP Lab) IRCCS Ospedale Policlinico San Martino Genoa Liguria Italy; ^4^ Department of Mathematics (DIMA) University of Genoa Genoa Liguria Italy; ^5^ Department of Health Sciences University of Genoa Genoa Liguria Italy; ^6^ Nuclear Medicine Unit AOU Città Della Salute e Della Scienza di Torino Turin Piedmont Italy; ^7^ Department of Medical Sciences University of Turin Turin Piedmont Italy; ^8^ Department of Pharmacy and Biotechnology Alma Mater Studiorum ‐ University of Bologna Bologna Emilia‐Romagna Italy; ^9^ Regional Neuroscience Department Genoa Liguria Italy; ^10^ Istituto Nazionale di Fisica Nucleare Genova Italy

**Keywords:** Alzheimer's disease, amyloid positron emission tomography (PET), arterial density, cerebrovascular architecture, perivascular clearance

## Abstract

**INTRODUCTION:**

Regional patterns of amyloid beta (Aβ) deposition in Alzheimer's disease (AD) may be influenced by cerebrovascular architecture. We examined the relationship between normative arterial and venous density maps and cortical Aβ burden.

**METHODS:**

Seventy‐four amyloid‐positive AD patients underwent amyloid positron emission tomography (PET) and magnetic resonance imaging. Regional Aβ uptake was quantified; spatial associations with normative arterial (time‐of‐flight magnetic resonance angiography) and venous (susceptibility‐weighted imaging) density maps were assessed using correlation analyses and partial least squares (PLS) regression, controlling for early‐frame PET as a proxy for perfusion.

**RESULTS:**

Higher arterial density was associated with lower Aβ uptake (*r* = −0.68, *p* < 0.001), independent of venous density and perfusion. PLS explained 71% of regional variance, highlighting temporo‐limbic regions. Subject‐level analyses showed heterogeneous vascular–amyloid coupling, related to education and global Aβ burden.

**DISCUSSION:**

Arterial architecture may contribute to regional amyloid vulnerability through vascular clearance mechanisms.

## Highlights

- Normative arterial density maps predict regional amyloid burden in AD.
- Amyloid deposition is inversely related to arterial, more than venous, vascular density.
- Arterial–amyloid coupling persists after accounting for perfusion‐like PET signal.
- Multivariate modeling highlights temporo‐limbic regions as vascularly vulnerable.

## 1 BACKGROUND

Alzheimer's disease (AD) is pathologically defined by the accumulation of amyloid beta (Aβ) plaques and neurofibrillary tangles, along with neuroinflammation and neuronal loss.^1^ Among core diagnostic biomarkers,^2^ amyloid positron emission tomography (PET) allows for in vivo mapping of Aβ deposition, which reveals a characteristic cortical pattern; however, the biological determinants that shape the regional vulnerability to such pathological deposition remain incompletely understood.

Beyond Aβ production, growing evidence implicates Aβ clearance mechanisms as determinants of its regional buildup.^3^, ^4^, ^5^ In the healthy brain, interstitial Aβ is eliminated through enzymatic degradation, active transport across the blood–brain barrier (BBB), and drainage along perivascular routes into cerebrospinal fluid (CSF) or lymphatic channels.^6^, ^7^ Impairment in any of these pathways can lead to Aβ retention in vulnerable regions, and many studies indicate that sporadic AD arises predominantly from reduced clearance rather than excess production.^3^, ^8^ Among the clearance routes, cerebrovascular systems play a pivotal role. Arteries and arterioles serve not only as conduits for blood flow but also as anatomical highways for interstitial solute removal, a process known as intramural peri‐arterial drainage (IPAD).^4^, ^9^, ^10^ With aging or vascular pathology, arterial stiffening, loss of pulsatility, and endothelial dysfunction impair the perivascular drainage system, promoting Aβ deposition along vascular routes. In parallel, the glymphatic and meningeal lymphatic systems contribute to Aβ clearance. Together, these pathways form a vascular‐linked clearance system whose failure may influence amyloid accumulation in AD.^5^, ^6^, ^7^

The concept of neurovascular unit further supports the idea that vascular factors play a role in the pathogenesis of AD.^11^ BBB dysfunction, microvascular inflammation, and chronic hypoperfusion have been observed early in the disease course.^12^, ^13^ The neurovascular hypothesis suggests that vascular pathology is a driver of amyloid and tau accumulation, contributing to the progression of AD.^8^, ^14^

Given this background, an emerging question is whether regional variability in vascular architecture could help explain the regional vulnerability to amyloid deposition. Neuropathological and imaging studies have shown that Aβ frequently localizes around blood vessels and that areas with reduced perfusion or BBB disruption often overlap with sites of higher amyloid burden.^1^, ^13^

Building on these insights, our study aims to investigate whether normative maps of vascular density correlate with the cortical distribution of Aβ in AD.

By integrating vascular architecture with molecular imaging, this work provides an in vivo framework to test the vascular‐clearance hypothesis of AD and explore how cerebrovascular health may shape both the spatial and temporal trajectories of amyloid pathology.

**RESEARCH IN CONTEXT**

1. **Systematic review**: We reviewed experimental, neuropathological, and imaging literature on cerebrovascular contributions to amyloid clearance in AD, including IPAD and related perivascular mechanisms. Although prior studies suggested that impaired vascular clearance promoted amyloid accumulation, in vivo investigations linking vascular architecture to regional amyloid distribution are limited.
2. **Interpretation**: In this study, we examined whether normative cerebrovascular density maps were associated with the cortical distribution of amyloid burden in AD. Our results indicate an inverse spatial relationship between amyloid uptake and arterial density, which persists after accounting for perfusion‐like effects. These findings are consistent with a possible role of vascular architecture in shaping regional amyloid vulnerability, while not establishing a direct mechanistic link.
3. **Future directions**: Future research should assess whether individual‐level vascular imaging and functional measures of vascular health refine these associations and clarify their relevance across disease stages and in relation to tau pathology.

## 2 METHODS

### 2.1 Participants

We retrospectively included 74 patients with a diagnosis of AD from the in‐house PET/magnetic resonance imaging (MRI) database of IRCCS Ospedale Policlinico San Martino (Genoa, Italy). Participants were diagnosed according to clinical National Institute on Aging‐Alzheimer's Association criteria,^15^ with biomarkers interpreted in the context of contemporary research frameworks emphasizing a biological definition of AD.^2^ All patients were evaluated between January 2017 and December 2024 in accordance with institutional protocols. Inclusion criteria were (i) a clinical syndrome consistent with AD with symptom duration < 2 years at diagnosis, (ii) amyloid‑positive PET, and (iii) availability of a structural MRI suitable for PET registration, acquired on average within a year of the PET scan. Exclusion criteria were severe medical illness, major psychiatric disorders, contraindications to MRI, moderate to severe white matter hyperintensities (Fazekas ≥ 2^16^; large territorial infarcts, strategic lacunes, or brain tumors. Neurological and neuropsychological assessments were performed according to standardized local procedures.^17^

### 2.2 Image acquisition

#### 2.2.1 T1 MRI

High‐resolution MRI was used for PET registration and cortical parcellation only. A subset of 17/74 patients underwent in‐house 3T MRI using a three‐dimensional (3D) T1‐weighted magnetization‐prepared rapid gradient‐echo (MPRAGE) sequence. The remaining 57 patients had external 1.5T MRI 3D scans acquired in clinical practice. Because these examinations were acquired at multiple institutions, complete acquisition parameters were not always available. In this study, MRI data were used only to extract structural T1‐weighted images for anatomical segmentation and cortical parcellation.

#### 2.2.2 Amyloid PET imaging

Subjects underwent amyloid PET/computed tomography (CT) on a Siemens BioGraph 16 scanner. All fluorinated tracers available during the study period were included ([^18^F]flutemetamol, [^18^F]florbetapir, [^18^F]florbetaben), following recommendations from the manufacturers and the European Association of Nuclear Medicine/Society of Nuclear Medicine and Molecular Imaging guidelines.^18^ Images were reconstructed on a 256×256 matrix (1.33×1.33×2.0 mm voxels). Injected doses ranged from 185 to 370 MBq. Amyloid positivity was established by two experienced nuclear medicine physicians with more than 10 years of experience in nuclear neuroimaging through independent visual assessment of the PET scans, blinded to clinical information; in cases of discordant interpretations, the final classification was determined by consensus. When feasible, a dual‐phase protocol was used: Immediately after tracer injection, a perfusion‐like early static frame (5 min for florbetapir; 10 min for flutemetamol; 0 to 5 or 0 to 10 min for florbetaben^19^, ^20^, ^21^) was acquired (data available for 50 patients). Early‐phase images represent a proxy of relative tracer delivery and perfusion‐like effects and correlate with [^18^F]FDG uptake.^19^, ^22^, ^23^, ^24^

To be clear, throughout this paper, “amyloid PET” refers to the conventional late‐frame acquisition used to assess amyloid burden, whereas “early frame” refers to the early post‐injection perfusion‐like acquisition used as a proxy for cerebral blood flow.

#### 2.2.3 Image processing

**FIGURE 1:**
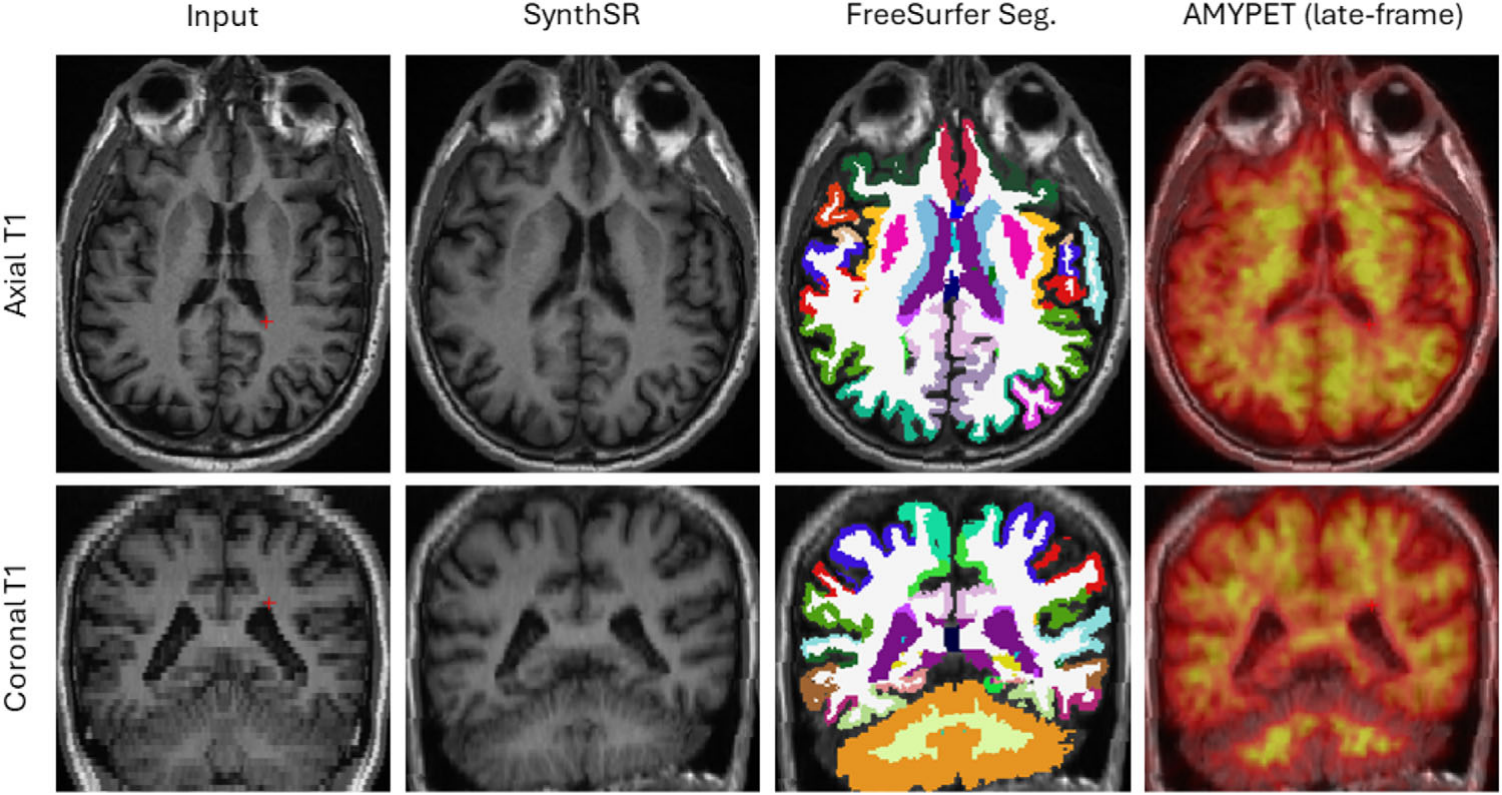
Example of synthetic MRI reconstruction and multimodal co‐registration. Representative example of an externally acquired T1‐weighted MRI (3‐mm of axial slice thickness) on the left, and the corresponding synthetic 1‐mm isotropic MPRAGE‐like reconstruction generated using SynthSR. The Desikan‐Killiany cortical parcellation obtained from FreeSurfer processing and the co‐registered late‐frame amyloid PET image are shown as overlays. The first row displays axial views and the second row coronal views.

Because MRI data obtained externally were highly heterogeneous, we reconstructed synthetic 1‐mm isotropic MPRAGE‐like volumes using SynthSR^25^ to standardize inputs (see Figure 1 for a representative example). Native and synthetic T1 images were then processed with FreeSurfer (version 7.4.1, cross‐sectional stream) for skull stripping, bias correction, cortical surface reconstruction, and Desikan‐Killiany (DK) parcellation.^26^

A visual quality control (QC) of the brain masks, surfaces, and parcellations was performed. Eleven external MRI scans were excluded due to insufficient slice coverage leading to unreliable cortical segmentation.

T1 MRI images and PET scans were non‐linearly registered to the ICBM 2009c non‐linear symmetric template (https://nist.mni.mcgill.ca/icbm‐152‐nonlinear‐atlases‐2009/; ^27^, ^28^ (see Figure 1 for an example of MRI‐PET co‐registration). A second QC step assessed T1‐PET alignment in Montreal Neurological Institute (MNI) space using overlap percentage (<90% flagged as suspicious) and centroid distance (>15 mm flagged). Flagged scans were reprocessed until convergence by adjusting co‐registration parameters.

After T1‐PET co‐registration, cortical standardized uptake value ratios (SUVRs) were computed using whole cerebellum as reference. Regional SUVRs were extracted in DK cortical regions of interest (ROIs) (34 per hemisphere).

Image processing and QC were performed by a medical physicist with over 6 years of experience in PET/MRI neuroimaging analysis.

#### 2.2.4 Normative vascular density maps

To approximate the brain's vascular architecture, we used atlas‐aligned normative vascular density maps^29^ from independent healthy‐adult datasets (42 individuals): (i) an arterial density map, derived from 3T time‐of‐flight magnetic resonance angiography (TOF‑MRA), processed to extract arterial signal and projected onto the cortical surface and (ii) a venous density map derived from 3T susceptibility‐weighted imaging (SWI), processed to enhance venous contrast and segment venous structures.

In the normative vascular density maps, MRI data were acquired on a 3T scanner: Acquisition parameters included a multi‐band TOF‐MRA sequence (voxel size 0.625 × 0.625 × 1.3 mm; repetition time [TR]/echo time [TE] = 23/3.6 ms) and a multi‐echo SWI sequence (voxel size 0.6 × 0.6 × 1.2 mm; TR = 28 ms; TE = 6.9/12.6/18.3/24.0 ms). Vessel enhancement in the source atlas was performed using a Frangi‐based multiscale filtering approach prior to segmentation, as described in the original methodological work and in the atlas publication.^29^, ^30^ Voxel‐wise arterial and venous probabilistic density maps were then generated by averaging binarized vessel maps across participants in MNI space, resulting in population‐level maps reflecting the spatial probability of vascular structures across the cortex.

Both vascular maps were provided already parcellated into the 68 cortical DK regions, providing regional density values directly comparable to our PET‐derived SUVRs. No additional preprocessing or spatial registration of the vascular maps was required (see Figure S1 for TOF and SWI density maps). Conceptual details on TOF^31^ and SWI^32^ contrast mechanisms are provided in the Supplementary Materials.

### 2.3 Statistical analyses

#### 2.3.1 Group‐level analyses

For each cortical ROI (*n* = 68), we extracted regional amyloid uptake (also for early frames) and vascular density (TOF, SWI).

ROI‐wise Pearson correlations were computed between regional amyloid and both arterial and venous density maps. To assess independence between vascular components, partial correlations were computed by residualizing one vascular map from the other (e.g., TOF|SWI). Additional partial correlations adjusted for early‐frame amyloid to account for perfusion‐driven tracer delivery.

Because cortical maps exhibit a strong spatial autocorrelation, statistical significance of correlations and partial correlations was assessed using a spherical rotation (“spin‐test”) with 5000 random rotations of the ROI centroids. Empirical two‐tailed *p* values reflected the proportion of permuted correlations exceeding the observed value.

Multivariate associations between vascular density (X = TOF, SWI) and amyloid uptake (Y) were modeled using PLS with two latent components. Outputs included ROI‐level scores, component weights, predicted amyloid values, and explained variance in X and Y. Component 1 (PLS 1) maps were visualized on cortical surfaces and ranked bar plots.

#### 2.3.2 Subject‐level analyses

For each subject, we computed region‐wise correlations between amyloid PET uptake and the two vascular maps, TOF and SWI (amyloid PET and early‐frame separately). Correlations were Fisher z‐transformed. Confounding variables (education, age, Mini‐Mental State Examination [MMSE], global cortical SUVR) were evaluated for collinearity using variance inflation factor (VIF).

Interindividual variability was assessed using kernel‐density distributions of z‐values. TOF versus SWI correlations were plotted in the 2D coupling plane, one for each clinical variable. K‐means clustering (k = 2 to 4, silhouette‐based selection) was applied separately to each clinical variable, and cluster membership was projected in the TOF‐SWI plane. Cluster covariance ellipses and centers were shown for visualization.

The linear association between amyloid‐TOF and amyloid‐SWI correlations was quantified using ordinary least squares (OLS). A non‐parametric 95% bootstrap confidence band (5000 resamples) was estimated by refitting the model at each iteration. To quantify independent clinical effects, separate OLS models predicted amyloid‐SWI and amyloid‐TOF correlations using all clinical variables simultaneously (education, age, MMSE, global cortical SUVR). Models were fitted independently for amyloid PET and early‐frame PET.

A 2D primary component analysis was applied to the matrix of subject‐level amyloid–vascular correlations. Primary component (PC) 1 represented the dominant covariation axis between TOF and SWI coupling; PC2 captured variability orthogonal to PC1. Explained variance was reported separately for late‐ and early‐frame amyloid.

All tests were two‐tailed with *α* = 0.05 unless otherwise specified.

## 3 RESULTS

We included 74 amyloid‐positive patients with AD (mean age 71.7 ± 6.3 years; 51.4% female; median education 12 ± 4.1 years; baseline MMSE 25.0 ± 3.6).

### 3.1 Group‐level analysis

**FIGURE 2:**
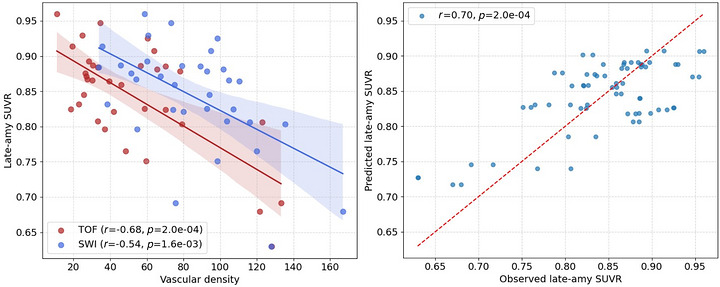
Left: Regional association between amyloid uptake and vascular density. Scatterplots show the inverse relationship between cortical amyloid burden (mean SUVR) and vascular density across cortical regions, using arterial (TOF) and venous (SWI) normative maps. Analyses were restricted to the left hemisphere, given the symmetry of vascular density templates and comparable hemispheric amyloid levels. Right: Observed versus predicted regional amyloid uptake (late‐frame PET). Scatterplot showing the correspondence between observed regional SUVR values and those predicted by the partial least squares model integrating TOF and SWI density maps. The diagonal identity line is shown in red.

At the ROI level, amyloid uptake showed a robust negative association with vascular density across cortical regions. This effect was markedly stronger for the arterial map derived from TOF (*r* = −0.68, *p* < 0.001) than for the venous map derived from SWI (*r* = −0.54, *p* < 0.001), indicating that regions with lower vascular density tend to accumulate more amyloid (Figure 2, left).

Because TOF and SWI maps were moderately correlated across regions (*r* = 0.61, *p* < 0.001), we assessed their independent contributions. When controlling for venous density, the amyloid association with TOF remained significant (partial *r* = −0.44, *p* = 7.0 × 10^−3^), whereas the reciprocal analysis showed that the SWI association lost significance once TOF was accounted for. Similarly, controlling for early‐frame PET signal attenuated but did not eliminate the association between late‐frame amyloid and TOF (partial *r* = −0.35, *p* = 2.7×10^−^^2^), suggesting that arterial architecture contributes explanatory value beyond perfusion‐like effects.

**FIGURE 3:**
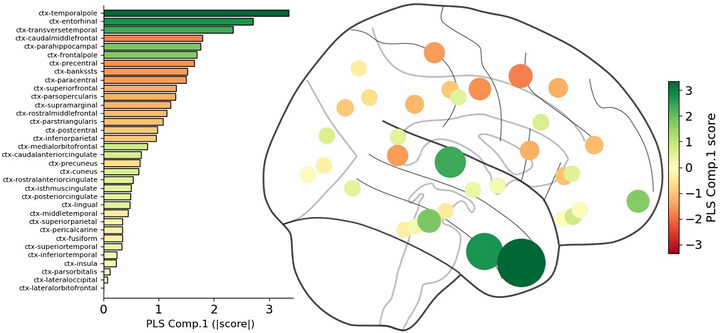
Spatial projection of PLS component scores on the cortical surface. Each sphere represents a cortical region plotted at its MNI centroid. Sphere colour indicates the direction of the regional loading, and sphere size reflects its magnitude. The strongest positive loadings cluster within temporal and limbic territories, indicating that these regions contribute most prominently to the inverse vascular–amyloid association captured by the multivariate PLS model. PLS, partial least squares.

A multivariate PLS model integrating both vascular maps significantly predicted regional amyloid distribution (*r* = 0.70, *p* < 0.001; Figure 2, right). The first latent component alone explained 47% of the variance in amyloid uptake, with the second adding only a negligible increment. Spatial projection of PLS loadings onto a glass brain revealed that temporal regions, including entorhinal, parahippocampal, transverse temporal, and temporal pole cortices, were the strongest contributors to the latent amyloid–vascular pattern (Figure 3). These areas showed large positive loadings, consistent with the overall negative association: regions with denser vasculature exhibited lower amyloid burden. In contrast, frontal, occipital, and parieto‐central areas presented weaker, sometimes negative loadings, suggesting more heterogeneous or opposing contributions to this relationship.

Overall, group‐level findings indicate a dominant arterial component to the spatial coupling between vascular density and amyloid deposition, with venous architecture showing weaker and partly dependent associations. The pattern is anatomically organized, with temporal cortices exerting the greatest influence on the amyloid–vascular relationship.

### 3.2 Subject‐level analysis

At the single‐subject level, regional correlations between amyloid uptake and vascular density maps showed substantial interindividual heterogeneity (Figure S2).

**FIGURE 4:**
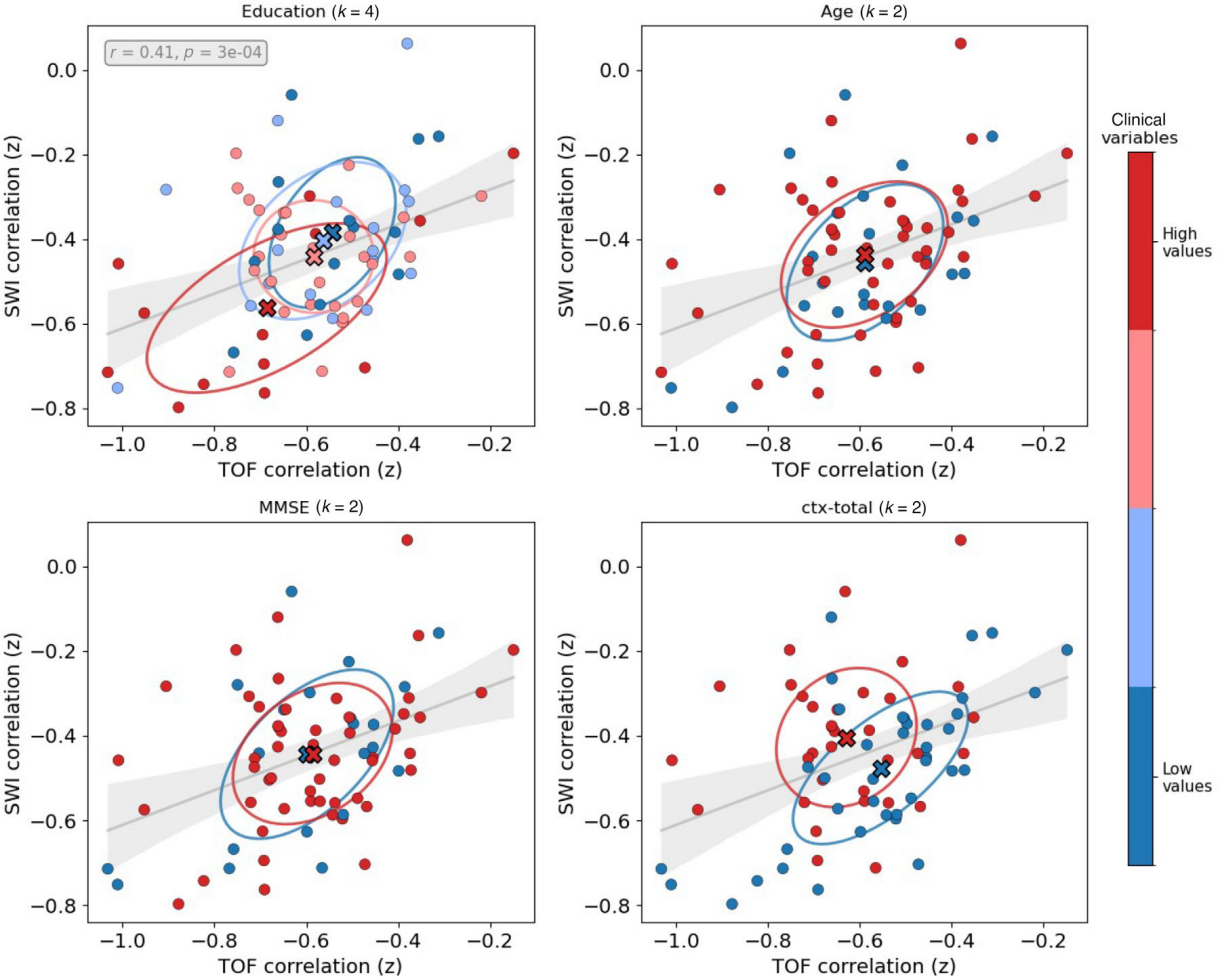
Subject‐level amyloid–vascular coupling from amyloid PET, plotted in the TOF‐SWI plane using z‐transformed correlations. Each point represents one subject. Panels show data‐driven clustering based on four clinical variables: education (top‐left), age (top‐right), MMSE (bottom‐left), and global cortical amyloid burden (ctx‐total, bottom‐right). For each variable, subjects were clustered using k‐means after median imputation and z‐scoring, with the optimal number of clusters (k = 2‐4) determined via silhouette maximization. Cluster membership is indicated by point colour, ellipses represent cluster covariance in the TOF‐SWI space, and centroids are marked with “×”. A low‐opacity regression line (grey) with a 95% bootstrap confidence band (5,000 resamples) illustrates the linear relationship between arterial and venous coupling. Correlation statistics are reported in the inset of the top‐left panel. *mini mental state examination (MMSE)*.

Joint analysis of TOF and SWI correlations revealed a coherent multivariate structure with the primary axis of variability (PC1, 71% of variance), reflecting a shared arterial–venous coupling pattern (Figure 4). Education and global cortical amyloid burden emerged as the main factors modulating this structure, with education associated with stronger vascular–amyloid coupling and higher amyloid burden associated with weaker coupling. A more detailed description of the subject‐level analyses is provided in the Supplementary Materials (Supplementary Results).

Regression analyses confirmed that education (negative) and global amyloid burden (positive) significantly predicted vascular–amyloid coupling, explaining approximately 21% of the variance. Additional clustering analyses and visualizations are provided in Figure S3.

## 4 DISCUSSION

In this study, we investigated how normative cerebrovascular architecture, derived from atlas‐based arterial (TOF) and venous (SWI) density maps, related to the spatial distribution of amyloid burden in AD. Across analyses, we found that regions with denser vascularization consistently exhibited lower amyloid uptake, with a clear predominance of the arterial compartment. These findings indicate that macroscopic vascular organization may act as a structural determinant of regional vulnerability to amyloid accumulation and support a mechanistic link between vascular architecture and protein clearance.^3^, ^4^, ^5^, ^9^, ^33^

At the group level, both arterial and venous density showed robust inverse associations with amyloid uptake; however, arterial density accounted for a substantially larger proportion of the explained variance. Although TOF and SWI maps were moderately collinear, reflecting shared anatomical substrates, only the arterial association remained significant after controlling for venous density. Similarly, multivariate modeling using PLS demonstrated that arterial contributions dominated the primary component explaining regional amyloid distribution, whereas venous density provided only minor, non‐independent contributions. These converging results may indicate that arterial topology is the main vascular correlate of the cortical amyloid landscape.

Associations persisted after adjusting for early‐frame PET, a proxy of tracer delivery and perfusion‐like effects. This suggests that vascular density relates to longer‐term clearance processes rather than tracer delivery. Together, these findings reinforce the notion that structural vascular features provide explanatory value beyond hemodynamic effects.

The cross‐sectional design and use of normative vascular maps of the present study prevent conclusions about the directionality of the observed associations. Importantly, the vascular density maps derived from independent cohorts rather than patient‐specific vascular measurements. Therefore, the associations identified here reflect the spatial correspondence between regional amyloid burden and the normative organization of the cerebrovascular network, rather than vascular remodeling occurring within the studied individuals. Future studies combining subject‐specific vascular imaging and longitudinal amyloid biomarkers could clarify the temporal and mechanistic relationships between vascular architecture and amyloid accumulation.

While the present correlation‐based approach does not directly probe clearance mechanisms, the observed spatial patterns are consistent with established models of cerebrovascular‐mediated amyloid removal, including IPAD pathway.^9^ The efficiency of IPAD depends on arterial pulsatility, wall integrity, and the spatial extent of peri‐arterial drainage routes.^4^, ^10^ Within this framework, regions characterized by a richer and more compliant arterial architecture may support more effective amyloid clearance. This offers a mechanistic interpretation for the observed inverse association between arterial density and regional amyloid burden.

Beyond IPAD, the glymphatic system, driven by CSF flux along peri‐arterial spaces, facilitates interstitial amyloid removal and operates synergistically with peri‐arterial drainage, particularly during sleep.^5^, ^34^ Prior works suggested that regions with richer arterial networks – through enhanced pulsatility and expanded perivascular space volume – are associated with more effective glymphatic function.^6^, ^7^, ^10^ Dysfunction across these interconnected pathways may promote amyloid accumulation and accelerate pathology.^3^, ^35^

Additional processes, including perivascular immune mechanisms, may further modulate amyloid clearance.^36^, ^37^ Accordingly, regional amyloid vulnerability may reflect not only vascular geometry but also the local efficiency of perivascular immune mechanisms.

Our findings align with the broader vascular hypothesis of AD, which posits that microvascular dysfunction and BBB breakdown contribute to amyloid and tau pathology.^8^, ^14^ Impaired arterial clearance may represent an early vascular insult linking systemic and cerebral processes.^4^, ^5^ Consistent with this view, impaired perivascular drainage has been proposed as a shared pathogenic mechanism between AD and CAA.^38^ Therapeutic strategies aimed at modulating arterial pulsatility, vasomotion, or CSF–interstitial fluid exchange may therefore hold promise for reducing amyloid burden.^3^, ^8^, ^10^, ^38^

Conversely, venous density contributed little to the spatial variance of amyloid burden, consistent with evidence that venous and dural lymphatic routes primarily facilitate bulk CSF drainage rather than direct removal of parenchymal waste.^4^, ^9^, ^10^ This arterial–venous asymmetry underscores the directional nature of perivascular clearance and further supports the preferential role of arterial topology in shaping amyloid distribution.

Knowledge regarding the vascular localization of Aβ derives mainly from neuropathological studies of CAA, a condition that frequently co‐occurs with AD. These studies indicate that when vascular amyloid deposition is present, it most commonly involves small arteries and arterioles.^33^ Amyloid PET tracers have been validated against neuropathology and primarily reflect fibrillar Aβ deposition in the brain parenchyma.^39^, ^40^ Although vascular amyloid may contribute to the signal, it cannot be reliably distinguished from parenchymal amyloid in vivo.^41^

Within this framework, our findings should be interpreted as reflecting spatial associations between vascular architecture and regional amyloid distribution rather than evidence regarding the specific vascular compartment of amyloid deposition.^33^

Recent ultra‐high‐field MRI studies demonstrate substantial interindividual variability in arterial patterns,^42^ arguing against the existence of a single canonical vascular template. In this context, normative, population‐derived vascular atlases represent a practical and biologically meaningful compromise for group‐level modeling of disease‐relevant spatial variance. Such anatomical variability may also constitute a substrate for individual susceptibility to amyloid accumulation, as suggested by evidence linking hippocampal vascular supply patterns to vascular reserve.^43^

The spatial pattern highlighted by the PLS model – encompassing entorhinal, parahippocampal, temporal pole, and transverse temporal cortices – closely overlaps with regions known to exhibit early vulnerability in AD. These areas combine high metabolic demand, complex vascular geometry, and early involvement in tau pathology, suggesting that local vascular architecture may confer both metabolic support and susceptibility to impaired clearance.^1^, ^3^, ^5^, ^10^, ^44^ The observed coupling between vascular density and amyloid burden in hippocampal regions further highlights the complexity of this relationship, as regions with dual or redundant arterial supply may exhibit distinct clearance dynamics and resilience patterns.^44^ Ex vivo ultra‐high‐field reconstructions further indicate that vessel‐rich entorhinal and perirhinal subfields display early phosphorylated tau and transactive response DNA binding protein 43 kDa pathology, underscoring the role of local vascular microenvironments in shaping sites of pathological initiation.^45^

Beyond group‐level organization, single‐subject analyses revealed structured variability in amyloid–vascular coupling. Subject‐specific arterial and venous correlations covaried along a dominant axis explaining most of the variance, indicating a shared vascular signature underlying individual differences. Clinical variables modulated this structure: Higher education and lower global amyloid burden were associated with stronger vascular constraints on amyloid distribution. This suggests that, early in the disease or in individuals with higher cognitive reserve, amyloid deposition remains more tightly coupled to vascular architecture, whereas increasing global amyloid burden may weaken this spatial dependence through saturation effects. Although the underlying mechanisms cannot be directly inferred, the observed influence of education is consistent with cognitive reserve acting as a proxy marker of cerebral resilience rather than a direct biological driver.^46^, ^47^

Normative vascular atlases may therefore offer a reference for contextualizing amyloid PET patterns. By providing a stable structural baseline, they enable assessment of whether an individual's amyloid distribution aligns with expected regional vascular support or deviates in ways that may signal clearance vulnerability. Integrating vascular priors into clinical or trial pipelines could refine staging, reduce regional variability, and enhance sensitivity to treatment effects. These concepts are consistent with converging evidence that vascular dysregulation and early BBB alterations are integral features of sporadic AD and interact closely with both amyloid and tau pathology.^12^, ^13^

Several limitations should be acknowledged. The normative vascular maps used here capture stable population‐level anatomical constraints; they do not reflect interindividual variability.^42^ Incorporating subject‐specific vascular imaging would make it possible to test whether individual deviations from normative patterns better explained within‐subject amyloid distributions.^32^, ^48^ Additionally, this study focused on structural vascular metrics, while functional measures of vascular reactivity were not assessed. Finally, MRI resolution limits may affect segmentation precision, although harmonization procedures mitigate this concern.^25^

Future work integrating individual vascular imaging, tau PET, and perfusion metrics across disease stages will be essential to clarify the temporal sequence linking vascular alterations to amyloid accumulation. By linking vascular topology, clearance physiology, and amyloid distribution, our findings support an emerging view in which preserving cerebrovascular health may be central to modifying AD progression.

## CONFLICT OF INTEREST STATEMENT

The authors declare no conflicts of interest. Author disclosures are available in the Supporting Information.

## CONSENT STATEMENT

All participants provided written informed consent for the use of clinical and imaging data. The study was approved by the local ethics committee and conducted in accordance with the Declaration of Helsinki.

## Supporting information




Supporting Information

Supporting Information
