# Supplementary material for: Exploring the spatial covariance of cerebral vascular density and amyloid burden in Alzheimer's disease
Source: Alzheimers Dement (Amst). 2026 Jun 18;18(2):e70390. doi: 10.1002/dad2.70390 (PMC13279348; doi:10.1002/dad2.70390)
Supplement: Supplementary file 1 — Supporting Information [file DAD2-18-e70390-s001.docx]

# **Supplementary material**

**Supplementary Methods**

Conceptually, TOF‑MRA highlights flowing arterial blood via inflow enhancement (i.e., unsaturated blood entering the imaging volume), supporting non‑contrast angiographic depiction of arteries (1), whereas SWI enhances venous conspicuity by exploiting magnetic susceptibility differences (particularly from deoxygenated blood) through processing that combines magnitude with filtered phase information, yielding strong venographic contrast suitable for venous structure mapping (2).

References

1. Shin T. Principles of magnetic resonance angiography techniques. **Investig Magn Reson Imaging.** 2021;25(4):209-217. doi:10.13104/imri.2021.25.4.209
2. Haacke EM, Mittal S, Wu Z, Neelavalli J, Cheng YC. Susceptibility-weighted imaging: technical aspects and clinical applications, part 1. **AJNR Am J Neuroradiol.** 2009;30(1):19-30. doi:10.3174/ajnr.A1400

## **Supplementary Results**

## **Subject-level analysis**

At the single-subject level, regional correlations between amyloid uptake and vascular density maps showed substantial interindividual heterogeneity. Fisher-z transformed correlation distributions revealed a broad range of coupling strengths for both TOF and SWI (**Supplementary Figure S2**). While TOF and SWI peaks overlap partially, the distributions remain distinct, highlighting that arterial contributions dominate, whereas venous effects are weaker.

Joint visualization of subject-specific TOF and SWI correlations in the TOF-SWI plane revealed a coherent multivariate structure (**Figure 4**). For amyloid load, unsupervised clustering and PCA demonstrated that the primary axis of variability (PC1, 71% of variance) aligned with a diagonal TOF-SWI covariation pattern. Education emerged as the dominant factor explaining separation along this axis, whereas global cortical amyloid burden drove separation along the secondary orthogonal axis (PC2, 29% of variance). A significant linear trend between TOF and SWI correlations (r = 0.41, P value = 3.0×10⁻⁴) was confirmed with bootstrap-derived 95% confidence intervals.

Regression analyses testing clinical modifiers showed that vascular coupling with amyloid was significantly predicted by education (negative) and global cortical amyloid burden (positive), explaining about 21% of the variance. Individuals with higher education showed stronger vascular-amyloid correlations, whereas those with higher amyloid load exhibited weaker negative coupling. The joint distribution of subject-level arterial and venous amyloid–vascular coupling across clinical stratifications is further illustrated in **Supplementary Figure S3.**


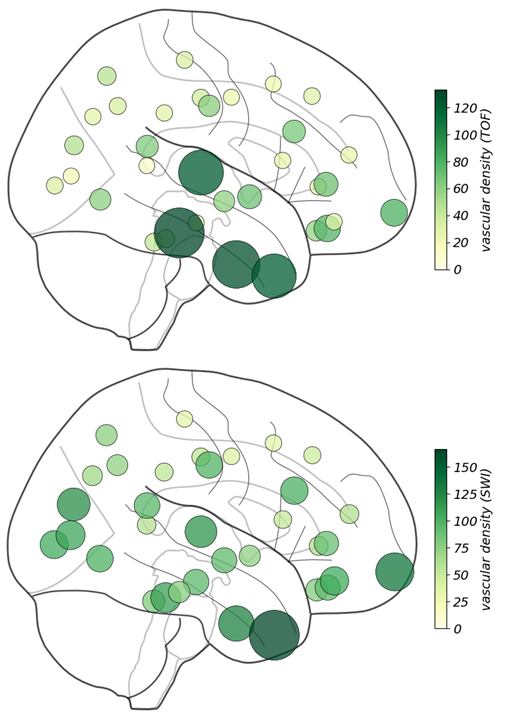


**Figure S1. Regional vascular density maps derived from TOF and SWI imaging.**Sagittal glass-brain projections showing the spatial distribution of cortical vascular density across the across the 68 Desikan-Killiany cortical regions (34 per hemisphere). The upper panel shows arterial density derived from TOF angiography, while the lower panel shows venous density derived from susceptibility-weighted imaging (SWI). Node size and color reflect the magnitude of regional vascular density across regions.


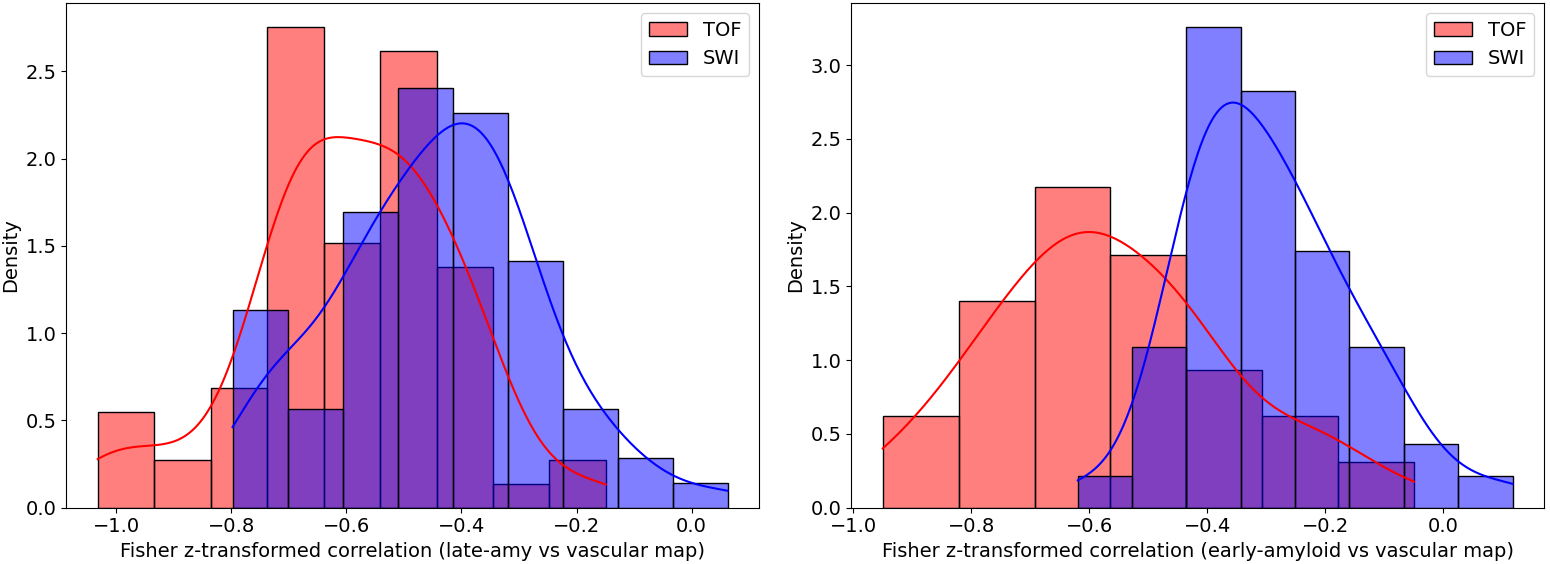


**Figure S2. Subject-specific vascular-amyloid correlations.**Distributions of subject-specific vascular-amyloid correlations for late (left panel) and early-frame PET (right panel). Histograms show z-transformed correlations between regional cortical amyloid uptake and normative vascular density maps. Both distributions are shifted below zero, reflecting an overall inverse relationship between vascular density and amyloid burden across subjects.


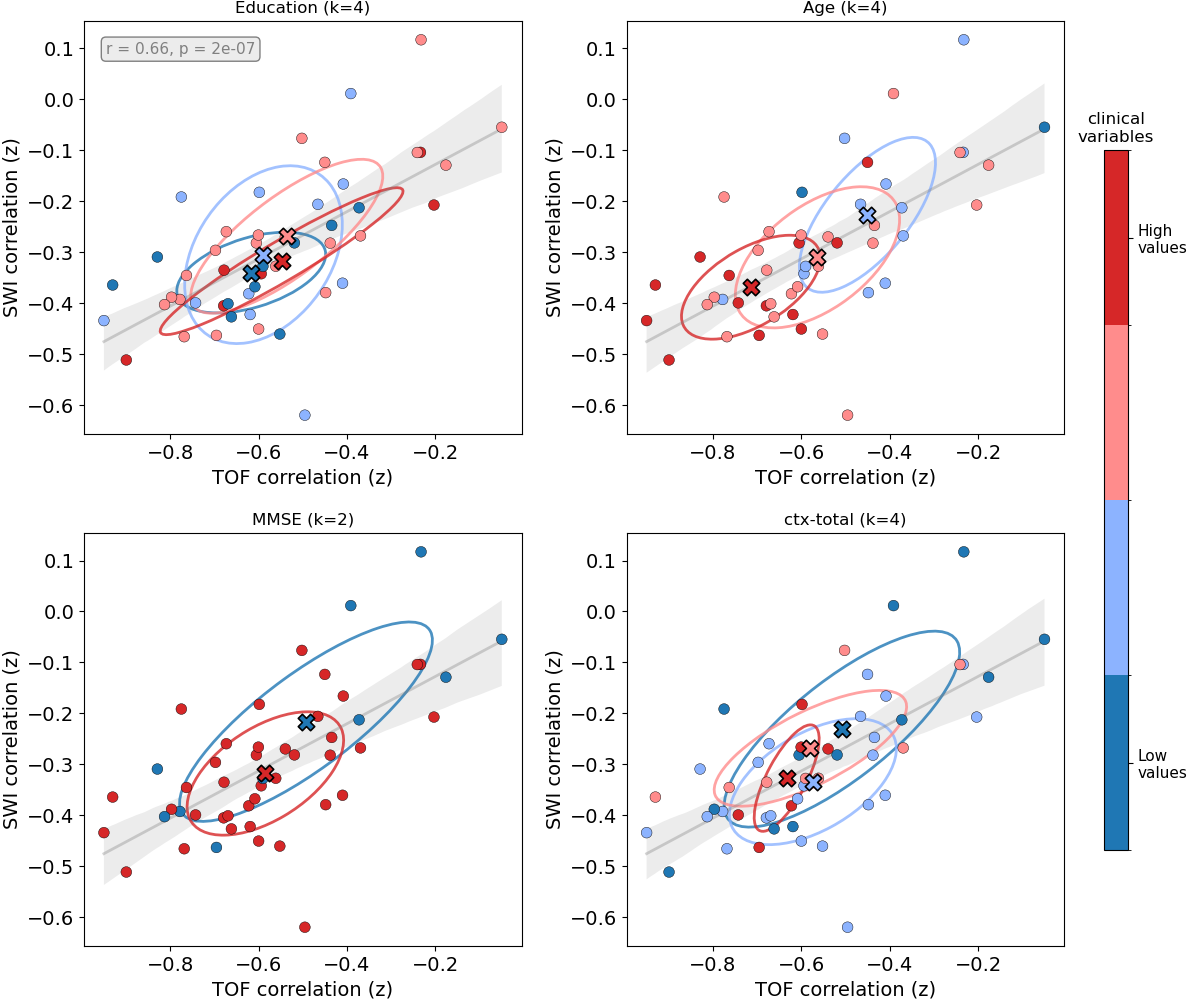


**Figure S3. Subject-level amyloid-vascular coupling.**Subject-level amyloid-vascular coupling from amyloid PET, plotted in the TOF-SWI plane using z-transformed correlations. Each point represents one subject. Panels show data-driven clustering based on four clinical variables: education (top-left), age (top-right), MMSE (bottom-left), and global cortical amyloid burden (ctx-total, bottom-right). For each variable, subjects were clustered using k-means after median imputation and z-scoring, with the optimal number of clusters (k = 2-4) determined via silhouette maximization. Cluster membership is indicated by point colour, ellipses represent cluster covariance in the TOF-SWI space, and centroids are marked with “×”. A low-opacity regression line (grey) with a 95% bootstrap confidence band (5,000 resamples) illustrates the linear relationship between arterial and venous coupling. Correlation statistics are reported in the inset of the top-left panel.
